# Intelligent Fusion Imaging Photonics for Real-Time Lighting Obstructions

**DOI:** 10.3390/s23010323

**Published:** 2022-12-28

**Authors:** Hyeonsu Do, Colin Yoon, Yunbo Liu, Xintao Zhao, John Gregg, Ancheng Da, Younggeun Park, Somin Eunice Lee

**Affiliations:** 1 Department of Electrical & Computer Engineering, Biomedical Engineering, Applied Physics, Biointerfaces Institute, Macromolecular Science & Engineering, University of Michigan, Ann Arbor, MI 48109, USA; 2 Department of Mechanical Engineering, University of Michigan, Ann Arbor, MI 48109, USA

**Keywords:** intelligent robotics, intelligent autonomous vehicles, intelligent drones

## Abstract

Dynamic detection in challenging lighting environments is essential for advancing intelligent robots and autonomous vehicles. Traditional vision systems are prone to severe lighting conditions in which rapid increases or decreases in contrast or saturation obscures objects, resulting in a loss of visibility. By incorporating intelligent optimization of polarization into vision systems using the iNC (integrated nanoscopic correction), we introduce an intelligent real-time fusion algorithm to address challenging and changing lighting conditions. Through real-time iterative feedback, we rapidly select polarizations, which is difficult to achieve with traditional methods. Fusion images were also dynamically reconstructed using pixel-based weights calculated in the intelligent polarization selection process. We showed that fused images by intelligent polarization selection reduced the mean-square error by two orders of magnitude to uncover subtle features of occluded objects. Our intelligent real-time fusion algorithm also achieved two orders of magnitude increase in time performance without compromising image quality. We expect intelligent fusion imaging photonics to play increasingly vital roles in the fields of next generation intelligent robots and autonomous vehicles.

## 1. Introduction

Intelligent robots and autonomous vehicles have rapidly emerged in recent years as the boundaries of current human capabilities have expanded [1,2,3]. The ability for vision systems to dynamically detect under challenging lighting conditions is essential for advancing intelligent robots and autonomous vehicles. Currently, most state-of-the-art methods to address challenging lighting conditions utilize passive optical components and computational processing [4,5,6,7,8,9,10,11,12,13,14,15,16,17,18,19,20]. Although these methods are relatively mature, computational approaches alone perform poorly under challenging lighting conditions when the signal-to-noise ratio is low, while passive optical components, such as filters and coatings, are limited in adaptability to dynamic and severe lighting scenarios. Rapid increases and decreases in contrast and saturation occlude objects, impairing vision with potentially severe consequences. Challenging lighting conditions are routinely addressed using polarization [9,12,20]. The intensity *I*_0_ at the image location *x,y* as a function of polarization *θ* is given by

(1) $$I_{0}\left(x,y\right)=I_{IF}\left(x,y\right)+\sum_{n}I{\left(x,y\right)}_{n}cos{\left(\theta_{n}\right)}^{2}$$

where intensity *I_IF_*(*x,y*) is free of lighting obstructions, *n* is the number of polarizations, and *I_n_*(*x,y*) is the polarization intensity. By rotating the polarization, lighting obstructions at each polarization according to Equation (1) can be physically removed. However, traditional approaches to rotate the polarization by mechanical manners are subject to beam deviations and spatial errors [21]. Simple approaches to rotate the polarization by predetermined increments also cannot readily adapt to dynamic scenarios. Furthermore, approaches identifying a single optimal polarization do not take into account the fact that different locations within an image may have different optimal polarizations.

**Figure 1:**
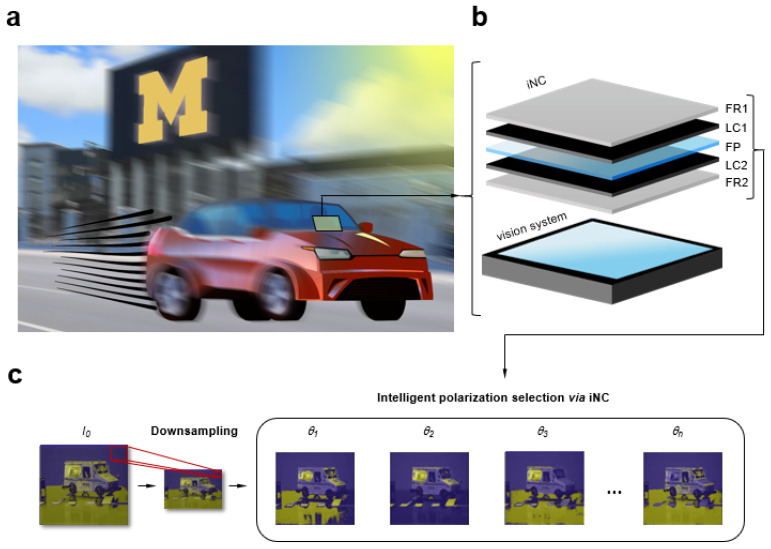
Intelligent fusion imaging photonics for real-time lighting obstructions. (**a**) Conceptual schematic of dynamic detection in challenging lighting conditions. Rapid increase and decrease in contrast and saturation obscures objects, resulting in loss of visibility. (**b**) Conceptual schematic of experimental setup consisting of iNC incorporated on top of a vision system (CMOS camera). FR1, fixed retarder; LC1, liquid crystal retarder; FP, fixed polarizer; LC2, liquid crystal retarder; FR2, fixed retarder. (**c**) Intelligent polarization selection process by iNC: Voltage to the iNC was modulated. Images were downsampled and segmented based on optimal polarization by analyzing each pixel such that the relation of the intensity of the pixel to the segmentation was not in the spatial domain but in the polarization domain.

In this work, we incorporate intelligent optimization of polarization into vision systems using the iNC (integrated nanoscopic correction) (Figure 1). We introduce an intelligent real-time fusion algorithm to address challenging and changing lighting conditions. Through real-time iterative feedback, we rapidly assess the optimal polarization which is difficult to achieve with traditional methods. Image quality is quantitatively scored using peak signal-to-noise ratio (PSNR), structural similarity (SSIM), and root mean squared error (RMSE). A fused image with minimal of lighting obstructions can be dynamically reconstructed using a pixel-based weighting mechanism. We demonstrate that fused images created by intelligent polarization selection uncover subtle features of occluded objects. We show that intelligent real-time fusion algorithm can significantly increase time performance without compromising image quality. We anticipate intelligent fusion imaging photonics will advance the ability of intelligent robots and autonomous vehicles to dynamically detect and respond to dynamic scenarios.

## 2. Results and Discussion

**Figure 2:**
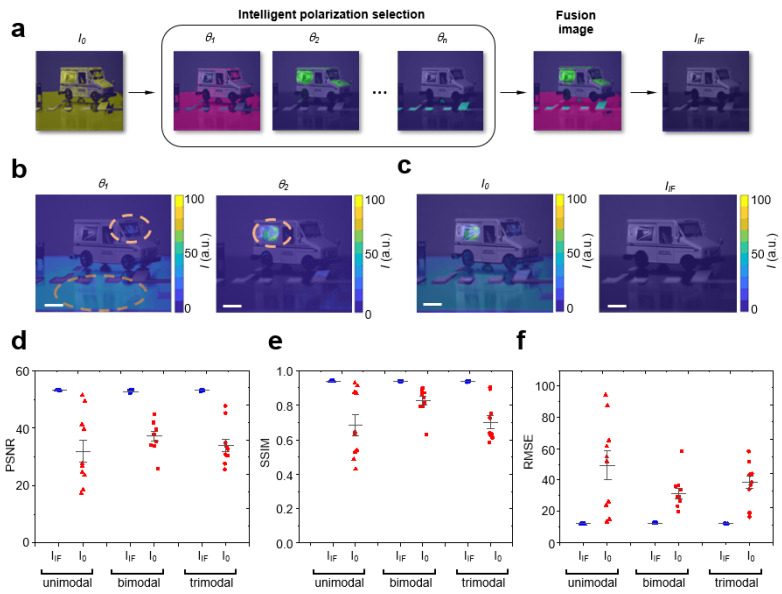
Fusion images by intelligent polarization selection uncovered subtle features of occluded objects. (**a**) Conceptual schematic of intelligent polarization selection and fusion imaging process. Intelligent real-time fusion (IF) algorithm determined optimal polarizations using iterative feedback. The fusion image was reconstructed by selecting the pixel value at the image index where the pixel-weight was the minimum on a per-pixel basis. I_IF_ was free of lighting obstructions. (**b**) Model vehicle photograph overlaid with intensity map at optimal polarization *θ*_1_ = 125° and optimal polarization *θ*_2_ = 45° determined by the IF algorithm. Scale bar: 1.3 cm. (**c**) Model vehicle photograph overlaid with intensity map for *I*_0_ and *I_IF_*. Scale bar: 1.3 cm. (**d**) Graph peak signal-to-noise ratio (PSNR) for *I*_0_ (red color) and *I_IF_* (blue color) comparing number of optimal polarizations present: unimodal, bimodal, trimodal. (**e**) Graph structural similarity (SSIM) for *I*_0_ (red color) and *I_IF_* (blue color) comparing number of optimal polarizations present: unimodal, bimodal, trimodal. (**f**) Graph root mean squared error (RSME) for *I*_0_ (red color) and *I_IF_* (blue color) comparing number of optimal polarizations present: unimodal, bimodal, trimodal. In (**d**–**f**), ten datasets were used. Each dataset consisted of 9 images captured at different polarizations with $\theta_{resol}$ = 20°. Each datapoint was calculated by averaging 9 images for *I*_0_ (red color). Each datapoint was a fusion image for *I_IF_* (blue color).

We firstly incorporated the iNC (integrated nanoscopic correction) [21,22], developed previously for nanoscopic imaging [21,22,23,24,25,26,27,28,29,30,31,32,33,34,35], into a vision system (CMOS camera) to address challenging and changing lighting conditions. The iNC was comprised of a series of fixed and variable retarders for systematic voltage control and dynamic modulation of the transmission polarization. By modulating the voltage to the iNC from 3 V to 10 V, the output transmission intensity corresponded to different input polarizations between 0° to 180° (Figure S1). For real-time capabilities, the iNC operated with millisecond response time (40 ms) (Figure S2). In contrast to conventional approaches using predetermined polarizations [9,12,20], the intelligent real-time fusion algorithm dynamically modulated the iNC to determine the optimal polarization to physically remove lighting obstructions using iterative feedback (Methods). We segmented images based on polarization and analyzed each pixel such that the relation of the intensity of the pixel to the segmentation was not in the spatial domain but in the polarization domain. Pixel-based weights were then assigned in the intelligent polarization selection process where each pixel was evaluated. Finally, a new fusion image was generated from pixel-based weights selected at all pixels (Figure 2a). In this way, the iNC enabled intelligent polarization selection as compared to conventional predetermination of polarization.

To experimentally characterize lighting in a controlled manner, we firstly created a modeled environment consisting of a model vehicle and crosswalk (Figure 2b,c). The controlled environment facilitated systematic creation, modification and reproducibility of lighting conditions. Illuminating the modeled scene showed image *I*_0_ (Figure 2c) in which different locations within the image had different optimal polarizations. To efficiently determine optimal polarizations, we used the intelligent real-time fusion (IF) algorithm to acquire images using iterative feedback. At one optimal polarization *θ*_1_, lighting obstructions were visible on the crosswalk and vehicle windshield (Figure 2b). At a different optimal polarization *θ*_2_, lighting obstructions were present on the side of the vehicle. The fusion image *I_IF_* (Figure 2c) was dynamically reconstructed by selecting the pixel value at the image index where the pixel-weight was the minimum on a per-pixel basis. With lighting obstructions removed, the fusion image revealed hidden features of the vehicle, windshield, and crosswalk (Figure 2c). To quantitatively assess the fusion process, we constructed synthetic datasets recapitulating experiments in which lighting obstructions were added to ground truth images. As different locations within images may have different optimal polarizations as observed in experiments, we compared image quality of fusion images as a function of number of optimal polarizations present (unimodal, bimodal, trimodal). We quantitatively scored image quality using quantitative metrics: peak signal-to-noise ratio (PSNR), structural similarity (SSIM), and root mean squared error (RMSE). As the number of optimal polarizations present increased, fusion images showed significantly enhanced PSNR as compared to reconstructions identifying only a single optimal polarization (Figure S3). Fusion images reduced the mean squared error by two orders of magnitude on average, resulting in a PSNR increased by 20 with nearly zero variance (Figure 2d). Fusion images demonstrated that SSIM values on average exceeded 0.95 (Figure 2e) and the RMSE on average reduced by a factor of 5 (Figure 2f). These results provide evidence that fusion images dynamically generated by the IF algorithm significantly enhanced image quality.

**Figure 3:**
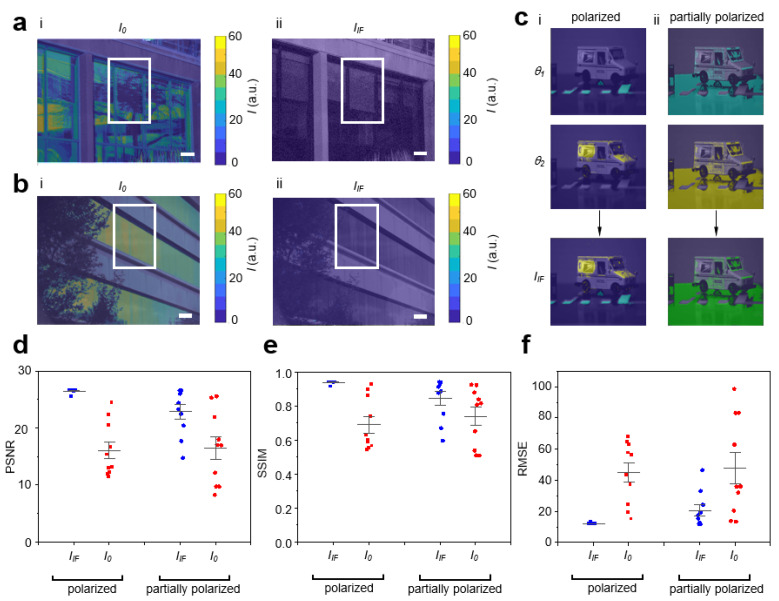
IF algorithm was robust for various lighting obstructions enabling classification. (**a**) Unsaturated lighting obstructions: Photograph of buildings with glass walls overlaid with intensity map for (**i**) *I*_0_ and (**ii**) *I_IF_*. Scale bar: 1 ft. (**b**) Saturated lighting obstructions: Photograph of buildings with glass walls overlaid with intensity map for (**i**) *I*_0_ and (**ii**) *I_IF_*. Scale bar: 1 ft. (**c**(**i**)) Polarized lighting obstructions: Conceptual schematic showing reconstructed intelligent fusion image *I_IF_*. (**c**(**ii**)) Partially polarized lighting obstructions: Conceptual schematic showing IF reconstructed intelligent fusion image *I_IF_*. (**d**) Graph of PSNR for *I*_0_ (red color) and *I_IF_* (blue color) comparing polarized versus partially polarized light obstructions. (**e**) Graph of SSIM for *I*_0_ (red color) and *I_IF_* (blue color) comparing polarized versus partially polarized light obstructions. (**f**) Graph of RSME for *I*_0_ (red color) and *I_IF_* (blue color) comparing polarized versus partially polarized light obstructions. In (**d**–**f**), ten datasets were used. Each dataset consisted of 9 images captured at different polarizations with $\theta_{resol}$ = 20°. Each datapoint was calculated by averaging 9 images for *I_0_* (red color). Each datapoint was a fusion image for *I_IF_* (blue color). Multiple optimal polarizations were present (bimodal).

Having constructed fusion images in the modeled environment, we proceeded to the outdoor environment (Figure 3a). Many previous methods commonly address specific lighting obstructions, requiring specific parameters of the illumination and vision system. In this work, we demonstrated IF was robust for various lighting obstructions, enabling classification. In outdoor scenes, we observed various lighting obstructions. In Figure 3a(i), specular reflections added unsaturated features (trees) to the scene of buildings with glass walls, occluding hidden objects. We used IF to efficiently select optimal polarizations, dynamically reconstruct a fusion image, and classify and remove unsaturated features. With unsaturated lighting obstructions removed, the fusion image revealed hidden objects (curtains) in the scene (Figure 3a(ii)). IF was also robust for saturated lighting obstructions (Figure 3b). In Figure 3b(i), specular reflections added saturated features (sun) to the scene and occluded hidden objects. Using IF to efficiently determine optimal polarizations and dynamically construct a fusion image, we classified and removed saturated features. The fusion image in Figure 3b(ii) revealed hidden objects (blinds) in the scene. As specular reflections can be also classified as polarized or partially polarized, we quantitatively characterized IF using synthetic datasets in polarized and partially polarized scenarios (Figure 3c). We quantitatively scored fusion image quality using PSNR, SSIM and RMSE. For polarized and partially polarized lighting obstructions, fusion images showed an increase in PSNR (Figure 3d) and SSIM (Figure 3e). Fusion images displayed a decrease in RMSE (Figure 3f) for polarized and partially polarized lighting obstructions. While image quality was higher for polarized lighting obstructions, these results support that IF also improves image quality for partially polarized lighting obstructions. As specular reflections are usually at least partially polarized, IF can be used to classify and remove various lighting obstructions.

**Figure 4:**
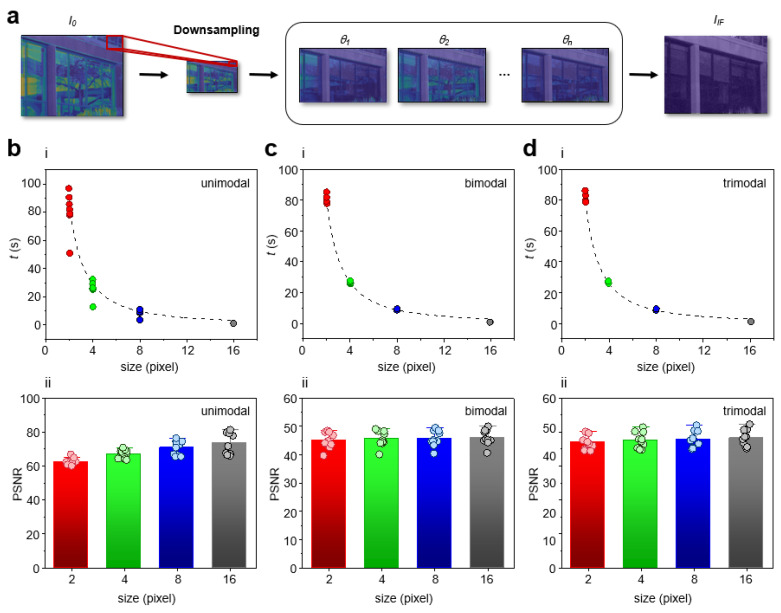
IF algorithm increased time performance without compromising image quality. (**a**) Conceptual schematic of the downsampling process in the IF algorithm. During the intelligent polarization selection process, the input dimensions of images were reduced. IF determined optimal polarizations using iterative feedback. The fusion image was reconstructed by selecting the pixel value at the image index where the pixel-weight was the minimum on a per-pixel basis. *I_IF_* was free of lighting obstructions. (**b**) Single optimal polarization present (unimodal): (**i**) Graph of time versus downsampling factor (red, green, blue, grey colors). (**ii**) Graph of PSNR versus downsampling factor (red, green, blue, grey colors). (**c**) Multiple optimal polarizations present (bimodal): (**i**) Graph of time versus downsampling factor (red, green, blue, grey colors). (**ii**) Graph of PSNR versus downsampling factor (red, green, blue, grey colors). (**d**) Multiple optimal polarizations present (trimodal): (**i**) Graph of time versus downsampling factor (red, green, blue, grey colors). (**ii**) Graph of PSNR versus downsampling factor (red, green, blue, grey colors).

Finally, we investigated the real-time performance of IF. In addition to efficiently determining optimal polarizations, a downsampling process was also implemented to further decrease memory and computational complexity of IF (Figure 4a). In outdoor scenes, lighting obstructions were observed, and the Image domain focused IF algorithm (Figure S4, Methods) was used to determine the optimal polarizations. During the intelligent polarization selection process, the input dimensions (3036 × 4024 pixels) of images were reduced to decrease memory and computational complexity for the purpose of capturing dynamic scenes at high speeds. To systematically study time performance, we varied input dimensions as a function of number of optimal polarizations present (Figure 4b–d). We observed an exponential relationship between the process time and the input dimensions, where the time for processing the set of images was approximately 1.03 s for downsampling factor of 16 (Figure 4b(i),c(i),d(i)). As a quantitative metric of image quality, the PSNR of the fusion images were calculated at different downsampling factors. (Figure 4b(ii),c(ii),d(ii)). We found that the change in PSNR with various downsampling factor was marginal. This finding is significant because the time cost can be reduced without compromising image quality. While the change in PSNR with various downsampling factor was marginal, increasing the downsampling factor indefinitely is such that lighting obstructions can no longer be distinguished. Thus, we considered a response time of 1.03 s (downsampling factor of 16) as the threshold for real-time and to maintain highest possible image quality and accuracy of the algorithm. For applications where millisecond high speed is a priority, the processing speed can be further reduced to milliseconds by further increasing the downsampling factor (response time 450 ms; downsampling factor 32).

A benefit of IF is the ability for real-time analysis enabled by intelligent optimization of polarization with the iNC in conjunction with downsampling. We have demonstrated IF can preserve image quality while simultaneously decreasing memory and computational complexity. This will allow for objects to be dynamically detected in challenging lighting scenarios. A limitation is IF is not compatible if scenes are completely unpolarized. However, as specular reflections are usually at least partially polarized, we expect this to be a rare but possible case. In the future, implementing complementary approaches, such as computational processing, together with IF can be used to address rare but possible unpolarized scenarios.

## 3. Conclusions

In summary, we have demonstrated an intelligent real-time fusion algorithm by incorporating intelligent optimization of polarization into vision systems using the iNC. We demonstrated that real-time iterative feedback with downsampling can significantly improve time performance without compromising image quality using quantitative metrics. We demonstrated fused images by intelligent polarization selection uncovered subtle features of occluded objects. We anticipate intelligent fusion imaging photonics to open new applications and capabilities of intelligent robots and autonomous vehicles in the future.

## 4. Methods

### 4.1. Experimental Setup

The iNC [21] was outfitted on top of a CMOS camera (Basler). Daq card (National Instrument) was used to relay signals from the computer to the camera and to the iNC according to Figure S1. Computation was conducted using MATLAB software (MathWorks) and images were collected in real-time using the IF algorithm. Computation was conducted in Python for time response analysis by incorporating a multithreading library to process multiple pairs of images at once. Python utilized Numpy and Pytorch, substituting the built-in MATLAB libraries.

### 4.2. IF Algorithm

The IF algorithm was developed to serve two different purposes: high image quality output and high response time. Image Fusion aimed to utilize the captured data to generate a high-quality image with minimal lighting obstructions. Iterative polarization can provide a high response time for utilization in dynamic scenarios. Thus, the IF algorithm applied two methods: Image Domain Focus Scanning (Figure S4) and Time Domain Focus Scanning (Figure S5), to create a versatile algorithm fitting different environments.

**Fusion:** Image Fusion utilized the available data to construct an image with minimal lighting obstructions. As the images captured by the iNC/CMOS were stored in memory, the images were compared to analyze the impact of lighting obstructions at different polarization angles on a per-pixel basis. This is possible because the images vary purely on the polarization domain and by capturing the images at different polarization angles, the image space was segmented independent of spatial relations. In our work, the image space was segmented up to $\lfloor \frac{180}{\theta_{resol}}\rfloor +1$ meaningful segments where $\theta_{resol}$ is the polarization resolution, and each segment represents the unobstructed scene (*I_IF_*) with the lighting obstructions at polarization $\frac{\pi }{2}+\theta_{resol}\;k$ in the image domain, where *k* is the segment index. If a region of the image was impacted by lighting obstructions, the variance of intensity of a pixel between each index should be between $I_{k}\;\cos^{2}\left(\theta_{optimal}-\frac{\theta_{resol}}{2}\right)<I_{k+1}<I_{k}\;\cos^{2}\left(\theta_{optimal}+\frac{\theta_{resol}}{2}\right)$ where *I_k_* is the previous image, *I_k+1_* is the current image, and $\theta_{optimal\;}$is the actual polarization angle of the lighting obstruction. As $\theta_{optimal\;}$was unknown, $\frac{\pi }{4}$ was used in place of $\theta_{optimal\;}$to support maximum variance. The algorithm utilized this intensity bound by initializing a binary validation mask, *M*_0_(*x*,*y*), with 1′s, and updated the validation mask using

(2) $$M_{i}\left(x,y\right)=\left\{\begin{array}{c} 1\;if\;I_{k}\cos^{2}\left(\frac{\pi }{4}-\frac{\theta_{resol}}{2}\right)<I_{k+1}<I_{k}\cos^{2}\left(\frac{\pi }{4}+\frac{\theta_{resol}}{2}\right)\;and\;M_{i-1}\left(x,y\right)=1\;\\ ,\\ 0\;,\;otherwise\; \end{array}\right.$$

After processing all of the captured images, the IF algorithm created an obstruction-free image by utilizing the weighting mechanism in the Intelligent polarization selection, where each pixel was weighted and the image index with minimum image weight was selected using $w=\sum R\left(x,y\right)\;M\left(x,y\right)\;where$

(3) $$R\left(x,y\right)=\left\{\begin{array}{c} I_{n+1}\left(x,y\right)-I_{n}\left(x,y\right),\;\;if\;|\left(I_{n+1}\left(x,y\right)-I_{n}\left(x,y\right)\right)|>T\\ 0,\;otherwise \end{array}\right.$$

$I_{n+1}\left(x,y\right)$ is the current image, $I_{n}\left(x,y\right)$ is the previous image, *T* is the pixel intensity threshold, *R*(*x*,*y*) is the difference matrix, and *M(x,y)* is the updated binary validation mask.

### 4.3. Intelligent Polarization Selection

Intelligent polarization selection aimed to compare a set of images and determine the impact of lighting obstructions. To achieve this, in a set of images, the IF algorithm compared the n^th^ captured image, *I_n_(x,y),* with the (n + 1)^th^ captured image, *I_n_*_+1_*(x,y)*, to determine whether the algorithm was improving the image quality, attempting to reach the maximum quality. This process was started by capturing an image at voltage index 0 and then capturing an image at voltage index 1. The change in image quality was calculated using a weighing mechanism. The image weight *w* was calculated for each image using Equation (3). As outdoor scenes are easily impacted by noise, *T* protected the algorithm from impacts of noise. The two weights, current and previous frame, were first compared with the minimum impact threshold of the image, *T_min_*, to determine whether the change in image quality was significant as when the polarization was at its peaks (both maximum and minimum) the rate of intensity change was much lower. In deciding the relative image quality of the two images, the image with the lower weight was considered to be higher quality.

**Image Domain Focused Scanning:** To gain a complete perspective of the image domain, the image domain was segmented into $\lfloor \frac{180}{\theta_{resol}}\rfloor +1$ or *K* segments and each segment are captured by the iNC/CMOS (Figure S4(i)). Two consecutive images were paired to calculate the relative validation matrix, *M_k_*(*x*,*y*), and the difference matrix, *R_k_*(*x*,*y*) in independent threads (Figure S4(ii)). When all of the images were processed, the validation matrix was updated to *M*(*x*,*y*) and the relative image weights were calculated and set as an (1 × *k*) array, *W* (*i*). (Figure S4(iii),(iv)). Then, the optimal image index was calculated using

(4) $$I=\underset{t}{\min}W_{t}\left(i\right)\;where\;\;W_{t}\left(i\right)=\sum_{i}W\left(i\right)\;$$

The fusion image was calculated using

(5) $$I\left(x,y\right)=I_{m}\left(x,y\right)where\;\;m=\underset{t}{\min}R_{t}\left(x,y\right),\;R_{t}\left(x,y\right)=\left\{\begin{array}{c} R_{t}\left(x,y\right)\;\;if\;M_{t}\left(x,y\right)=1\\ I_{max},\;otherwise \end{array}\right.$$

where *I_max_* is the maximum pixel possible intensity of the image, usually 255, per pixel basis and *I*(*x*,*y*) is the fusion image, and the iNC/CMOS re-captured the image at the optimal polarization at the original resolution (Figure S4(v)).

**Time Domain Focused Scanning:** In time restrictive scenarios, capturing the entire image domain can be difficult. Thus, the IF algorithm supported a time domain focused algorithm that is more responsive of the surroundings (Figure S5). Instead of capturing the whole image domain, the iNC/CMOS captured the first image using polarization index 0. Then the next image was captured at polarization index 1. The binary validation matrix was calculated along with the weights of the two images. If the weight of the current image was greater than the previous image, the directionality of the polarizer was inverted, and the next image was captured at polarization index 0. However, if the new image had a weight lower than the previous image, the polarizer continued to step in the same direction, capturing the next image at polarization index 2. This process stopped when there were *N* consecutive frames where the weight difference between images were below the impact threshold, *T_min_*. As the algorithm continuously updated the binary validation matrix and the weights, the fusion image was also updated after each frame, where if a pixel was valid, the fusion image pixel was updated using the pixel value with lower weight. The time domain focus scanning algorithm has a safeguard to protect against dynamic objects where if more than third of the validation matrix values turned to 0, all previous data were removed, and the process restarted.

### 4.4. Quantitative Metrics

Image quality was quantified using three quantitative metrics: PSNR, SSIM and RMSE. Firstly, PSNR, modification of MSE, was used as a metric because this metric focused on maximal lighting obstructions, as well as overall residual lighting obstructions. As PSNR focused on the maximal difference between the input and reference image, making this metric much more robust to noise and sensitive to errors. PSNR was calculated as

(6) $$PSNR\left(I{(}_{IF}\left(x,y\right),\;I\left(x,y\right)\right)=10\log_{10}\left(\frac{I_{MAX}{}^{2}}{MSE\left(I_{IF}\left(x,y\right),\;\;I\left(x,y\right)\right)}\right)$$

where $I_{MAX}$ is 255, $MSE\left(I_{IF}\left(x,y\right),\;I\left(x,y\right)\right)=\frac{\sum {\left(I{(}_{IF}\left(x,y\right)-I\left(x,y\right)\right)}^{2}}{N}$, and *N* is the number of pixels in the image. Secondly, SSIM was used as a metric because this metric is a perception-based model that considered image degradation. SSIM was calculated as

(7) $$SSIM\left(x,y\right)=\frac{\left(2\mu_{x}\mu_{y}+C_{1}\right)\left(2\sigma_{xy}+C_{2}\right)}{\left(\mu_{x}^{2}+\mu_{y}^{2}+C_{1}\right)\left(\sigma_{x}^{2}+\sigma_{y}^{2}+C_{2}\right)}$$

where $\mu_{x},\mu_{y},\sigma_{x},\;\sigma_{y},\sigma_{xy}$ are the local means, standard deviations, and cross-covariance for images *x*, *y* and $C_{1}={\left(0.01\;I_{MAX}\right)}^{2}$, $C_{2}={\left(0.03\;I_{MAX}\right)}^{2}$. Thirdly, RMSE provided a measure of the differences between the input and reference image. RMSE was calculated as

(8) $$RMSE\left(I_{IF}\left(x,y\right),\;I\left(x,y\right)\right)=\sqrt{\frac{\sum {\left(I_{IF}\left(x,y\right)-I\left(x,y\right)\right)}^{2}}{N}}$$

## Data Availability

Correspondence and requests for data should be addressed to S.E.L.

## Supplementary Materials

The following supporting information can be downloaded at: https://www.mdpi.com/article/10.3390/s23010323/s1.
