# Supplementary material for: Intelligent Fusion Imaging Photonics for Real-Time Lighting Obstructions
Source: Sensors (Basel). 2022 Dec 28;23(1):323. doi: 10.3390/s23010323 (PMC9824281; doi:10.3390/s23010323)
Supplement: Supplementary file 1 [file sensors-23-00323-s001.zip › sensors-2078616-supplementary.pdf]

Supporting Methods

Modeled environment

To experimentally characterize lighting in a controlled manner, we created a modeled environment consisting of a model vehicle and crosswalk. Paint, cardboard and reflective tape to make a realistic model of road markings and signs. The controlled environment facilitated lighting conditions to be controlled, modified and reproduced. For lighting, white LED lamps (black body equivalent temperature 4,000-5,800 K) were used as the light source. Radial angle markings were affixed underneath the modeled environment to serve as guides for lighting placement and the iNC-CMOS.

Synthetic dataset

To quantitative assess the fusion process, we constructed synthetic datasets, recapitulating experimental conditions, in which lighting obstructions were added to a ground truth image. First, an image was chosen to be used as the ground truth. Second, image segmentation using *K*-means segmentation was conducted with the number of segments being a random value between 0 and 10. For each source of lighting obstruction, one segment was chosen to be added with lighting obstruction using a polarization intensity, *A*, polarized at a random θ value between 0° and 180°. Finally, synthetic images were created following equation

$$I_0(x,y) = I_{IF}(x,y) + \sum_k A_k \, B_k(x,y) \, cos(\theta_k)^2 \quad (9)$$

where intensity $I_{IF}(x,y)$ is ground truth image, *k* is the segment index, A_n is the intensity of the lighting obstruction at index *k,* and $B_n(x,y)$ is the binary map marking the regions of the segment.

Supporting Figures

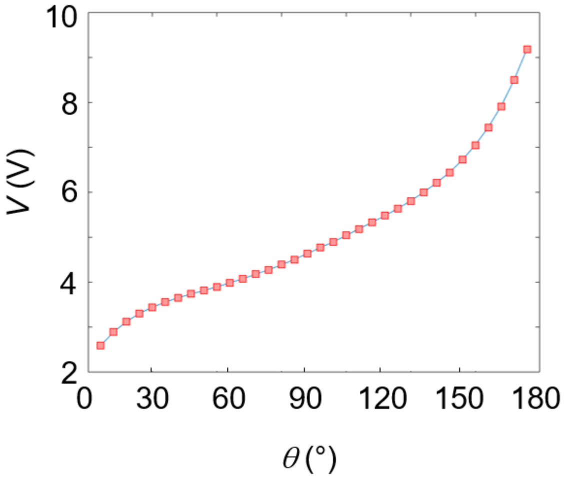

Figure S1. Graph of linear dependence of actuation voltage *V* as a function of transmission polarization θ.

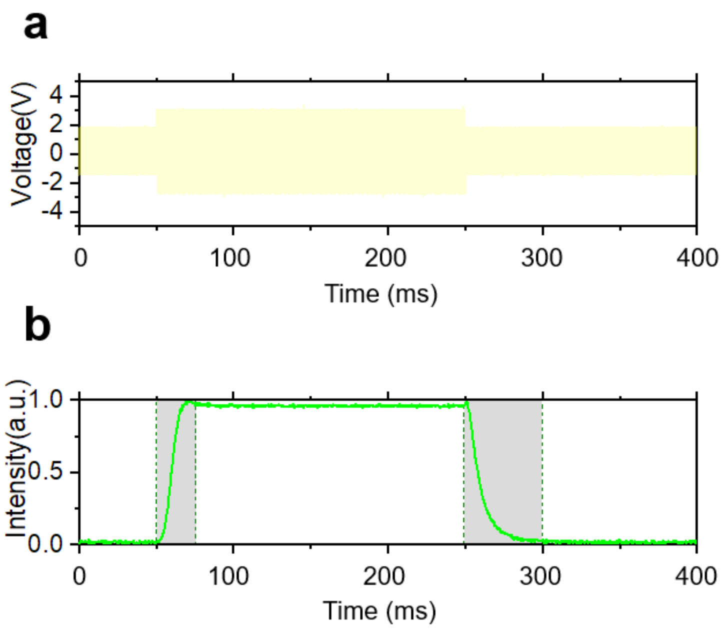

Figure S2. Millisecond response time characterization. (a) Graph of input voltage versus time. **(b)** Graph of output intensity versus time.

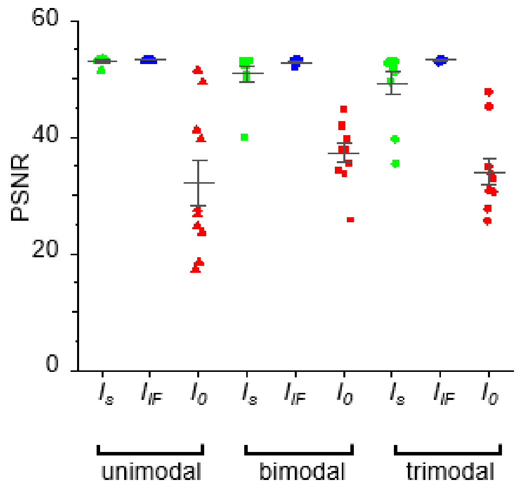

Figure S3. Intelligent fusion assessment. Graph peak signal-to-noise ratio (PSNR) for average captured images I_0 (red color), fusion image reconstructed using multiple optimal polarizations I_{IF} (blue color), and image constructed using only a single optimal polarization I_s (green color) as a function of number of optimal polarizations present: unimodal, bimodal, trimodal.

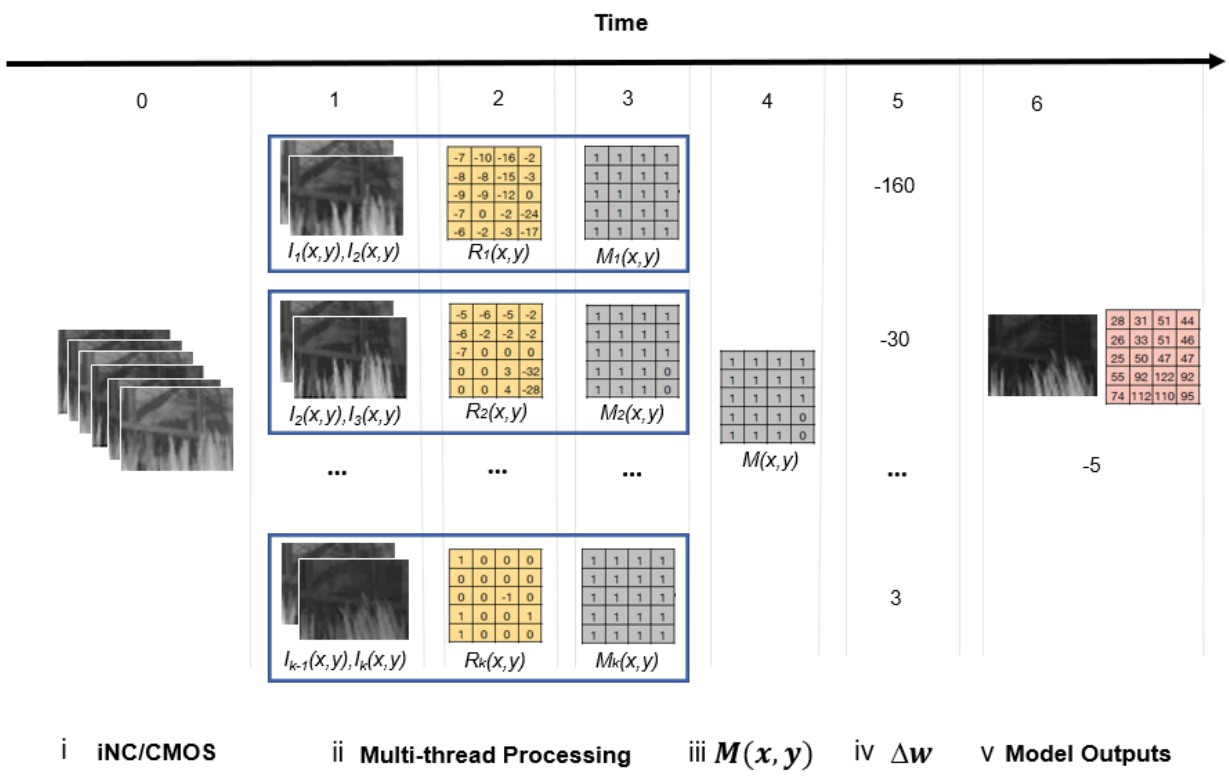

Figure S4. Image Domain Focused IF Algorithm Flowchart. . **(i) iNC/CMOS:** Dynamic outdoor scene photographs of grass illuminated by sunlight moving in the wind over time. All polarization images were captured in the initialization step. **(ii) Multi-thread Processing:** Consecutive image pairs were sent to different threads in the processer to conduct image analysis in parallel. First, two consecutive images were loaded to the thread, up to thread *k* where *k* is $\left\lfloor \frac{180}{\theta_{resol}} \right\rfloor + 1$. Second, the two images were compared to create a difference matrix, *R(x,y)*, following Eq. 3. The minimum impact threshold, T_{min}, was assumed to be 0. Lastly, the validation matrix, *M(x,y)*, was calculated based on the differences in the image. **(iii) Validation Mask:** A final *M(x,y)* was calculated by collecting the *K* partial validation matrix, $M_1(x,y)$, … , $M_k(x,y)$, and combining the invalidated regions, following Eq. 2. **(iv) Image weight:** Δw was calculated by taking the element-wise multiplication between the different matrix, *R(x,y)*, and validation matrix, *M(x,y)*. The weights were stored in a (1 x *k*) array with each thread storing the corresponding weight. **(v) Model Outputs:** The model output consisted of three parts: intelligent polarization selection, fusion image, and

optimal image. The intelligent polarization selection determined the optimal polarization utilizing the array of image weights, and calculated the index where the sum of the image weights up to the index was the minimum. The fusion image was created by first taking the base image, image at index 0, and updating the regions where the validation matrix, $M(x,y)$ is 1, using the minimum pixel values throughout different polarization. The optimal image was reported by taking the captured image at the chosen optimal polarization index.

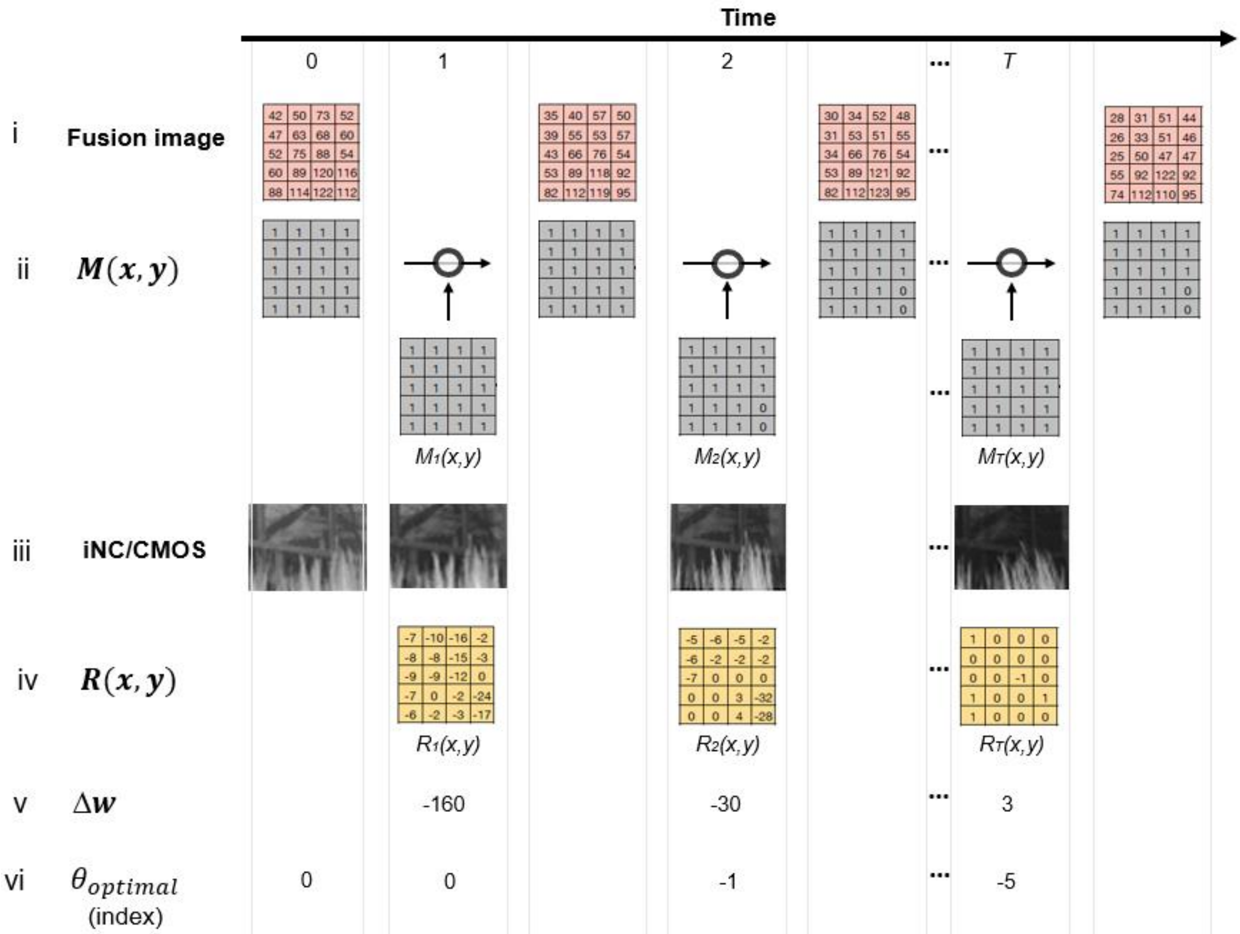

Figure S5. Time Domain Focused IF Algorithm flowchart. (i) Fusion Image: This image array was initialized by the base image. The values of the matrix were updated whenever a pixel value of the new image was lower than the base image and the validation matrix. **(ii) Validation Mask:** *M(x,y)* had a value of non-zero for that pixel. *M(x,y)* was initialized with 1's with the same dimension as the fusion image. Whenever a new image was taken, sanitization occurred following Eq. 2. **(iii) iNC/CMOS:** Dynamic outdoor scene photographs of grass illuminated by sunlight moving in the wind over time. Polarization image was captured at each time step. The first image, image at time t = 0 was set to the base image and the pixel values of the captured images were used for analysis. **(iv) Difference Matrix:** *R(x,y)* had the same dimension as the fusion image and was created after each image capture to take the difference between the fusion Image and the captured image. We assumed the minimum impact threshold, T_{min}, was set to 0. **(v) Image**

weight: Δw was calculated by taking the element-wise multiplication between the different matrix, *R(x,y)*, and validation matrix, *M(x,y)*. **(vi) Intelligent polarization selection:** The lower the weight, the better the polarization angle was optimized. The angular index for the optimal polarization angle was stored so the algorithm can determine the next polarization angle from which to sample.
